# Animal studies on glucagon-like peptide-1 receptor agonists and related polyagonists in nonalcoholic fatty liver disease

**DOI:** 10.1007/s42000-024-00541-2

**Published:** 2024-03-12

**Authors:** Chara Tsiampali, Ilias D. Vachliotis, Antonis Goulas, Stergios A. Polyzos

**Affiliations:** First Laboratory of Pharmacology, School of Medicine, Aristotle University of Thessaloniki, 54124 Thessaloniki, Greece

**Keywords:** Glucagon-like peptide-1 receptor agonists, Polypeptide, Animal studies, Nonalcoholic fatty liver disease, Nonalcoholic steatohepatitis

## Abstract

Nonalcoholic fatty liver disease (NAFLD) is a prevalent metabolic liver disease closely associated with the epidemics of obesity and type 2 diabetes mellitus (T2DM), but without licensed pharmacological treatment to date. As glucagon-like peptide-1 (GLP-1) receptor agonists (GLP-1RAs) are approved anti-diabetic and anti-obesity medications, they were also considered a potential therapeutic option for NAFLD. Preclinical studies suggest that GLP-1RAs have a beneficial effect on major NAFLD histological outcomes, i.e., hepatic steatosis and inflammation, through multiple intrahepatic mechanisms, including increased fatty acid β-oxidation, activation of autophagy, suppression of inflammation, and oxidative stress. Data on hepatic fibrosis are limited or inconclusive, although some studies reported improvement in indices of fibrosis or prevention of fibrosis initiation or reduction of collagen deposition. Whether the positive impact of GLP-1RAs on hepatic histology is indirect, i.e., through their action on extrahepatic tissues, or whether their action is direct, i.e., through activating GLP-1R on the hepatocytes, is still a controversial issue. Alongside GLP-1RAs, newly emerging peptide polyagonists (i.e., synthetic molecules that combine the amino acid sequences of more than one peptide, thus having the ability to bind more than one receptor) are now being investigated in NAFLD with high expectations. This review summarizes the existing knowledge derived from animal studies on the effects of GLP-1RAs and GLP-1RA related peptide polyagonists on NAFLD in an attempt to illuminate areas of uncertainty and provide the groundwork for future animal and clinical research in the field.

## Introduction

Nonalcoholic fatty liver disease (NAFLD) has become the most common chronic liver disease worldwide affecting more than one-third of the world population [1]. Mainly owing to its close association with the components of the metabolic syndrome, i.e., type 2 diabetes mellitus (T2DM), obesity, arterial hypertension and dyslipidemia, a change in the nomenclature of the disease to metabolic dysfunction-associated steatotic liver disease (MASLD) together with a change to the definition of the disease have been proposed [2]. Although benign in most cases, NAFLD may also evolve to more aggressive phenotypes, including nonalcoholic steatohepatitis (NASH) with or without fibrosis, cirrhosis, liver failure and hepatocellular carcinoma (HCC), which render NAFLD a serious public health concern, if considering the high global prevalence of the disease [3]. To date, only weight loss through lifestyle modifications can be recommended to patients with NAFLD, which although effective, is difficult to sustain in the long term [4]. Despite the above-mentioned disease burden, there is currently no available FDA- or EMA-approved medication for the disease, although numerous agents have been investigated in preclinical and clinical studies [5].

Glucagon-like peptide-1 (GLP-1) receptor agonists (GLP-1RAs) are analogs of human endogenous GLP-1, initially approved for the treatment of T2DM and, subsequently, for the treatment of obesity [6]. Their well-known and established action in reducing post-prandial glucose levels and promoting weight loss have also rendered GLP-1RAs a promising therapeutic option for NAFLD [6]. In addition, peptide polyagonists (i.e., synthetic molecules that combine the amino acid sequences of more than one peptide, thus having the ability to bind to more than one receptor), such as tirzepatide (LY3298176) [a dual GLP-1RA and glucose-dependent insulinotropic polypeptide (GIP) receptor agonist] have also been introduced into the management of metabolic diseases, including T2DM and obesity, and also are under investigation for NAFLD with high expectations [7].

In this review, we aimed to collect the best available evidence from animal studies on the effects of GLP-1RAs and GLP-1RA related peptide polyagonists on NAFLD in an attempt to illuminate areas of uncertainty and provide the groundwork for further animal and clinical research in the field.

## Animal studies on GLP-1RAs in NAFLD

A selection of available animal studies evaluating the effects of different GLP-1RAs on NAFLD is presented in Table 1 and is briefly discussed herein. Regarding exenatide, Trevaskis et al. demonstrated that administration of AC3174, an exenatide analog, to a high-trans-fat, high-fructose, high-cholesterol diet (HTFD)-induced NASH mouse model led to a reduction in body weight and fat mass as well as in liver and serum lipid levels and to improvement in hepatic steatosis and inflammation [8]; hepatic collagen-1 protein was also decreased, indirectly implying a potential for improvement of hepatic fibrosis; similar effects were observed after administration of AC3174 in a genetically obese mouse model lacking leptin (*ob/ob*), except for the improvement in hepatic collage-1 protein [8]. Notably, treatment of GLP-1 receptor knock-out mice with AC3174 had no effect on body weight or on the above-mentioned hepatic and plasma parameters [8], indicating the mediating effect of GLP-1 receptor on NAFLD-related parameters.

**Table 1:** Action of various GLP-1RAs on NAFLD histological features in animal studies

First author, year [Reference]*	Animal model of NAFLD	GLP-1RA	Improvement in steatosis	Improvement in inflammation	Improvement in fibrosis
Trevaskis, 2012 [8]	HTFD mice and HTFD *ob/ob* mice	AC3174 (exenatide analog)	Yes; Yes**	Yes; Yes**	N/A; N/A**
Fang, 2021 [9]	GAN diet mice and GAN diet *ob/ob* mice	Beinaglutide	Yes; Yes**	N/A; N/A**	N/A; N/A**
Ao, 2016 [10]	HFD SD rats	Liraglutide	Yes	Yes	N/A
He, 2016 [11]	HFD mice	Liraglutide	Yes	Yes	N/A
Tølbøl, 2018 [12]	AMLN diet mice and AMLN diet *ob/*ob mice	Liraglutide	Yes; No**	No; No**	No; No**
Zhu, 2018 [13]	HFD mice	Liraglutide	Yes	Yes	N/A
Daniels, 2019 [14]	HFCC diet SD rats	Liraglutide	Yes	Yes (lobular inflammation) No (hepatocellular ballooning)	N/A
Duparc, 2019 [15]	HFCC-CDX diet mice	Liraglutide	Yes	Yes	No
Hao, 2019 [16]	HFD mice	Liraglutide	Yes	Yes	N/A
Luo, 2019 [17]	HFD mice	Liraglutide	Yes	Yes	Yes
Ao, 2020 [18]	HFD SD rats	Liraglutide	Yes	Yes	N/A
Fang, 2020 [19]	HFD mice	Liraglutide	Yes	Yes	N/A
Han, 2020 [20]	HFD mice	Liraglutide	Yes	Yes	N/A
Jin, 2020 [21]	HFD Wistar rats	Liraglutide	Yes	Yes	N/A
Kojima, 2020 [22]	STZ and HFD mice	Liraglutide	Yes	Yes	No
Liu, 2020 [23]	*db/db* mice	Liraglutide	Yes	Yes	N/A
Fang, 2021 [24]	HFD mice	Liraglutide	Yes	Yes	No
Perakakis, 2021 [25]	AMLN diet mice	Liraglutide	Yes	Yes	No
Somm, 2021 [26]	MCD diet mice	Liraglutide	No	Yes	Yes (prevented initiation of fibrosis)
Ji, 2022 [27]	HFD mice	Liraglutide	Yes	N/A	Yes
Yu, 2023 [28]	HFD mice RORα-LKO	Liraglutide	Yes	N/A	N/A
Møllerhøj, 2022 [29]	GAN diet mice	Semaglutide	Yes	Yes	No
Pontes-da-Silva, 2022 [30]	HFD mice	Semaglutide	Yes	Yes	N/A
Reis-Barbarosa, 2022 [31]	HFD mice	Semaglutide	Yes	N/A	N/A
Niu, 2022 [32]	HFD mice	Semaglutide	Yes	Yes	Yes
Inia, 2023 [33]	FFD Ldlr -/- mice	Semaglutide	Yes	Yes	No

*References are sorted alphabetically according to the GLP-1RA (primarily) and the year of publication (secondarily)

**It refers to the first and second group of animals implemented in the same study, respectively

Abbreviations: *AMLN *amylin liver nonalcoholic steatohepatitis; *FFD* fast-food diet; *GAN*, gubra amylin; *HFCC* high-fat high-cholesterol high-cholic acid; *HFCC-CDX* high-fat high-cholesterol high-cholic acid diet with hydroxypropyl-β-cyclodextrin in drinking water; *HFD* high-fat diet; *HTFD* high-trans-fat high-fructose high-cholesterol diet; *Ldlr* low-density lipoprotein receptor; *MCD* methionine-choline deficient; N/A, not available; *RORα* retinoic acid receptor-related orphan receptor α; *SD* Sprague-Dawley; *STZ*, streptozotocin

Regarding beinaglutide, Fang et al. showed that it could reduce food intake, gastric emptying, body weight, liver function tests (LFTs), and glucose levels in a dose-dependent manner, thus improving hepatic insulin sensitivity and hepatic steatosis. The authors proposed that the action of beinaglutide may be associated with improvement of fatty acid (FA) β-oxidation and mitochondrial function [9].

Liraglutide was the GLP-1RA used in most animal studies (Table 1). Ao et al. examined the effect of liraglutide on NAFLD in a high-fat diet (HFD) Sprague–Dawley (SD) rat model for 4 weeks and reported improvement in hepatic steatosis, lobular inflammation, and ballooning. They also found that liraglutide may act, at least partly, through diminishing endoplasmic reticulum (ER) stress [10]. He et al*.* suggested that liraglutide improved serum lipid profile and hepatic steatosis by inducing autophagy through the adenosine monophosphate activated protein kinase (AMPK)/mammalian target of rapamycin (mTOR) pathway [11], which has a key role in autophagy modulation [34]. Other authors similarly noted that autophagy is related to the pathophysiology of NAFLD [35]. In another study, Tølbøl et al. employed a diet-induced obesity (DIO) NASH mouse model as well as *ob/ob* mice*,* which were treated with liraglutide, obeticholic acid (a farnesoid-X-receptor [FXR]), or elafibranor (a dual proliferator-activated receptor-a/δ [PPAR-α/δ]) for 8 weeks [12]. They showed that liraglutide reduced body weight, food intake, liver fat, and serum alanine aminotransferase (ALT) and aspartate aminotransferase (AST) in both models. Liraglutide also ameliorated steatosis in DIO, but not in *ob/ob* mice and had no effect on hepatocyte ballooning and fibrosis in both models [12]. The authors reported that liraglutide brought about changes in transcriptional pathways mainly associated with glucose metabolism [12]. Zhu et al., using a HFD-induced NAFLD mouse model, showed that body weight, LFTs, lipid profile, glucose, and insulin tolerance as well as hepatic steatosis were improved after 4 weeks of liraglutide treatment. The authors reported that the alleviation of NAFLD was related to the suppression of the Nod-like receptor protein amilypyrin domain containing 3 (NLRP3) inflammasome [13]. Daniels et al. investigated the efficacy of liraglutide in a rat NASH model. They demonstrated that liraglutide treatment reduced body weight and LFTs, improved hepatic steatosis, and decreased the severity of lobular inflammation, though it did not affect hepatocellular ballooning [14]. Duparc et al. evaluated the 2-week effect of liraglutide in a high-fat (60%), high-cholesterol (1.25%), and high-cholic acid (0.5%) along with hydroxypropyl-β-cyclodextrin (2%) in drinking water (HFCC-CDX) dietary mouse model of NASH and demonstrated that liraglutide decreased caloric intake and body weight and improved insulin resistance. Liraglutide was also shown to improve hepatic steatosis and inflammation, but not fibrosis [15]. Hao et al. showed that liraglutide improved hepatic steatosis and inflammation and suggested that hepatic lipid accumulation was reduced through stimulating AMPK, which downregulates the mTOR complex 1 (mTORC1)/sterol regulatory element binding protein 1 (SREBP1) signaling pathway [16]. Luo et al. evaluated the 10-week effect of liraglutide in a HFD mouse model, reporting improvement in hepatic steatosis, inflammation, and fibrosis [17]. This was partly achieved through downregulating the tumor necrosis factor-alpha (TNF-α) pathway [17], which seems to be implicated in the pathogenesis of NAFLD [36]. Ao et al. showed that 4-week liraglutide administration in a SD rat NAFLD model reduced body weight, food intake, and LFTs, leading to improvement in insulin resistance, hepatic steatosis, lobular inflammation, and hepatocellular ballooning. The anti-inflammatory effect of liraglutide was hypothesized to be partly associated with the suppression of mTORC1 signaling and NLRP3 inflammasome, basic elements of autophagy [18]. Fang et al. validated the beneficial effects of liraglutide on food intake, body weight, insulin resistance, and hepatic steatosis in HFD-fed mice and proposed that liraglutide treatment activated the autophagy-lysosomal-dependent lipid degradation [19]. They also noted that liraglutide restored autophagic balance mainly through restoring the transcription factor EB (TFEB)-mediated autophagy-lysosomal pathway [19]. In another study**,** Han et al*.* showed that the beneficial effects of liraglutide on the liver were achieved through stimulating the Sestrin2-mediated nuclear factor-erythroid 2-related factor-2 (Nrf2)/heme oxygenase-1 (HO-1) pathway, suggesting another potential mechanism via which liraglutide ameliorates NAFLD through enhancing the antioxidative potential of the liver cells [20]. Jin et al*.* confirmed the beneficial effects of liraglutide on LFTs, insulin resistance, hepatic steatosis, and inflammation, and they also reported that NAFLD activity score (NAS) and insulin resistance were positively correlated with resistin [21], an adipokine with proinflammatory action [37]. Kojima et al. similarly showed a beneficial effect of liraglutide on hepatic steatosis and inflammation and, interestingly, a potentially preventive effect against the progression of NAFLD-associated HCC [22]. Liu et al. confirmed the beneficial effects of liraglutide on hepatic steatosis and inflammation in a genetically diabetic mouse model (*db/db*) and suggested that they may partly be attributed to changes in the composition of intestinal microbiota [23], an hypothesis that was also supported by other investigators [38, 39]. Fang et al. also verified the beneficial effects of liraglutide on hepatic steatosis and inflammation in a HFD mouse model and hypothesized that these may be mediated by regulating ER stress, thus reducing apoptosis, in the pancreas and the liver [24]. Perakakis et al. compared the effects of liraglutide and elafibranor on hepatic parameters in a dietary NAFLD mouse model and suggested that both elafibranor and liraglutide reduced body weight and improved insulin sensitivity and NAS. Furthermore, liraglutide improved hepatic steatosis and inflammation, but not fibrosis [25]. Notably, liraglutide was shown to restore the concentrations of bile acids, glycogen metabolism by-products, and pentoses, thus facilitating glycogen utilization turnover and nucleic acid formation [25]. Somm et al. administered liraglutide for 4 weeks to methionine-choline deficient (MCD) diet mice, but they observed no effect on body weight and glycemic and lipid parameters and no improvement in hepatic steatosis [26], thus contrasting with most findings presented herein (Table 1). However, liraglutide exerted anti-inflammatory action by preventing the accumulation of C16 and C14 ceramide/sphingomyelin species and by affecting gut microbiota and prevented the initiation of fibrosis [26]. Consistently with most previously published findings, Ji et al*.* reported that the administration of liraglutide to HFD mice reduced body weight, glucose, LFTs, histologically-confirmed hepatic steatosis, and bridging fibrosis after 2 weeks of treatment. Although a 2-week treatment seems to be rather short for improving a hard endpoint like hepatic fibrosis, mechanistically, these beneficial effects of liraglutide were attributed, at least in part, to its ability to inhibit the activation of the receptor for the advanced glycation end products (RAGE)/nicotinamide adenine dinucleotide phosphate (NADPH) oxidase signaling pathway, which is involved in the formation of reactive oxygen species (ROS), a finding also confirmed in in vitro experiments in the same study [27]. Yu et al. employed a HFD-induced NAFLD mouse model with hepatocyte-specific ablation of the retinoic acid receptor-related orphan receptor α (RORα) (RORα-LKO) to demonstrate that liraglutide alleviated hepatic steatosis via RORα-dependent autophagy activation, thus highlighting another mechanism of autophagy activation by GLP-1RAs [28]. Of note, RORα are widely expressed nuclear receptors, which can modulate the transcription of several genes implicated in lipid metabolism in the liver [40].

There are also some relevant studies investigating the effects of semaglutide in animal models. Møllerhøj et al*.* showed that an 8-week semaglutide treatment in a gubra amylin (GAN) DIO NASH mouse model reduced food intake, body weight, and LFTs and improved lipid profile. As shown in most studies with other GLP-1RAs, hepatic steatosis and inflammation were improved, but not fibrosis. Notably, the authors suggested that semaglutide may reduce de novo collagen synthesis without causing degradation of pre-existing collagen fibers [29], implying that semaglutide may not improve existing fibrosis, but it may prevent the initiation and progression of fibrosis, which, however, needs other more focused studies to be definitively demonstrated. Pontes-da-Silva et al. showed similar effects of a 4-week semaglutide treatment in a HFD-induced NAFLD mouse model on food intake, body weight, and insulin resistance, as well as on hepatic steatosis and inflammation. The authors hypothesized that semaglutide improved steatosis by increasing FA β-oxidation and by decreasing hepatic glucose uptake and ER stress and that it improved inflammation by affecting the cytokine and adipokine profile [30]. Reis-Barbarosa et al. proposed that semaglutide may mitigate hepatic steatosis and hepatocellular ballooning in HFD-induced NAFLD mice through the modulation of the AMPK/mTORC1 signaling pathway [31], similarly to the above-mentioned effect of liraglutide [16]. Niu et al. showed that a 12-week administration of semaglutide in HFD-induced NAFLD mice reduced body weight, glucose, insulin resistance, LFTs, serum lipids, inflammatory markers [TNF-α, interleukin (IL)-6, IL-1β], and oxidative stress markers [malonaldehyde (MDA)], thus improving hepatic steatosis, inflammation, mitochondria damage, and fibrosis. Interestingly, metabolomic analysis in this study indicated that semaglutide treatment upregulated L-isoleucine and methacholine or downregulated L-histidinol, arachidonic acid, and 4,5- leukotriene (LT) A4 LysoPC(16:0), which are metabolites involved in the pathogenesis of NAFLD [32]. Inia et al. examined the effect of semaglutide on NAFLD in a fast-food diet (FFD) Ldlr -/- Leiden mice for 12 weeks, which reduced body weight, fat mass, LFTs, and serum lipids. Importantly, liver transcriptomic analysis demonstrated that semaglutide reduced the expression of genes involved in hepatic steatosis, inflammation, and fibrosis, which was confirmed histologically for steatosis and inflammation, but not for fibrosis [33]. However, further digital pathological analysis based on artificial intelligence (AI) indicated that semaglutide may not quantitatively affect fibrosis, but may improve collagen reticulation, i.e., prevent the formation of accumulated collagen fibers [33].

## Animal studies on GLP-1RA related peptide polyagonists in NAFLD

GLP-1 receptors are also common pharmacologic targets in combination with other peptide agonists in the setting of peptide polyagonists [41]. In this regard, the pleiotropic actions of two or three different receptor agonists may be additive but also complimentary to each other, thus being possibly more effective than GLP-1RAs in multifactorial diseases, including obesity, T2DM, and NAFLD [42]. The first peptide polyagonist that was officially approved for T2DM and obesity was tirzepatide (LY3298176), a dual GLP-1/GIP receptor agonist which is currently under investigation in RCTs for obesity and NAFLD [7]. Animal studies that have evaluated the effects of other novel peptide polyagonists in models of NAFLD are presented in Table 2 and briefly summarized hereby.

**Table 2:** Action of various GLP-1RA related peptide polyagonists on NAFLD histological features in animal studies

First author, year [Reference]	Animal model of NAFLD	Peptide polyagonists	Improvement in steatosis	Improvement in inflammation	Improvement in fibrosis
*GLP-1/ GCG dual receptor agonists*					
Valdecantos, 2017 [43]	MCD diet mice and HFD mice	G49	Yes; Yes*	Yes; Yes*	N/A; N/A*
Patel, 2018 [44]	HFD mice	Aib2 C24	Yes	Yes	Yes
Boland, 2020 [45]	AMLN diet mice and AMLN diet *ob/*ob mice	Cotadutide	Yes; Yes*	Yes; Yes*	Yes; Yes*
Nestor, 2022 [46]	AMLN diet mice	ALT-801	Yes	Yes	Yes
*GLP-1/ GIP dual receptor agonist*					
Liu, 2021 [47]	AMLN diet mice	CY-5	Yes	N/A	N/A
*GLP-1/ FGF-21 dual receptor agonist*					
Pan, 2020 [48]	HFD *ob/*ob mice	GLP-1-Fc-FGF-21 D1	Yes	Yes	N/A

*It refers to the first and second group of animals implemented in the same study, respectively

Abbreviations: *AMLN* amylin liver nonalcoholic steatohepatitis; *FGF-21* fibroblast growth factor-21; *GCG* glucagon; *GIP* glucose-dependent insulinotropic polypeptide; *GLP-1* glucagon-like peptide-1; *HFD* high-fat diet; *MCD* methionine-choline deficient; *N/A* not available

Most studies reported the effects of dual GLP-1/glucagon (GCG) receptor agonists in NAFLD animal models. Valdecantos et al. employed two NAFLD animal models (MCD diet and HFD mice), which underwent partial hepatectomy after treatment with G49, a dual GLP-1/GCG receptor agonist. G49 ameliorated histological features of NASH (steatosis and inflammation), reduced apoptosis and oxidative stress, improved mitochondria function and, importantly, increased survival by reducing the rate of postoperative liver failure and improving liver regeneration [43]. Patel et al. showed that administration of Aib2 C24, another dual GLP-1/GCG receptor agonist, alongside HFD feeding, prevented the development of hepatic steatosis, inflammation, and fibrosis in mice [44]. In another study, Boland et al. compared the effects of cotadutide (a dual GLP-1/GCG receptor agonist), liraglutide, and obeticholic acid in two mouse models of NAFLD [amylin liver NASH (AMLN) diet mice and AMLN diet *ob/ob* mice] to show that cotadutide outperformed other treatments in improving major histological endpoints, i.e., hepatic steatosis, inflammation, NAS, and fibrosis. Of note, the authors provided evidence that the hepatic effects of cotadutide were directly mediated through GCG receptor signaling, but not through direct GLP-1R signaling, which may be absent in the liver [45]. Similarly, Nestor et al. compared ALT-801 (another dual GLP-1/GCG receptor agonist), semaglutide and elafibranor in AMLN diet NAFLD mouse model to validate the beneficial effects of GLP-1/GCG dual agonism in NAFLD hepatic outcomes. In this study, ALT-801 was shown to be superior to semaglutide and elafibranor in improving hepatic steatosis and NAS score but inferior to elafibranor in reducing fibrosis stage [46]. Of note, ALT-801 is considered a balanced dual receptor agonist with comparable affinity for the GLP-1R and GCGR, in contrast to cotadutide, which exhibits higher affinity to GLP-1R than the GCG receptor (5:1) [46].

Other relevant polyagonists have also been examined in a few animal studies. CY-5, a novel GLP-1/GIP dual receptor agonist, was recently discovered by Liu et al., which showed efficacy in reducing serum lipids, LFTs, and biopsy-proven hepatic steatosis in an AMLN diet NAFLD mouse model [47]. Pan et al. constructed a novel GLP-1/fibroblast growth factor (FGF)-21 dual receptor agonist (GLP-1-Fc-FGF21 D1), which was evaluated in HFD *ob/ob* mice in comparison with either FGF-21 (c-FGF21 S1) or GLP-1RA (dulaglutide) monotherapy. They showed that GLP-1-Fc-FGF21 D1 performed better than both comparators in reducing liver steatosis, inflammation, and hepatocellular ballooning, indicating another potential dual-acting agent in the pharmacological pipeline of NAFLD [48].

## Discussion

**Fig. 1:**
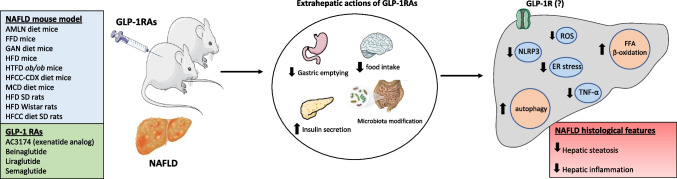
Hepatic effects of various GLP-1RAs in NAFLD animal models Treatment of diet-induced NAFLD mice or rats with GLP-1RAs has shown beneficial effects on major NAFLD histological outcomes, i.e., hepatic steatosis and inflammation. Data on hepatic fibrosis are inconclusive; although some studies reported improvement in indices of fibrosis or prevention of fibrosis initiation or reduction of collagen network complexity, most studies do not support a robust effect of GLP-1RAs on improving hepatic fibrosis. These beneficial effects of GLP-1RAs on hepatic histology have been attributed to multiple intrahepatic mechanisms, including increased FFA β-oxidation, activation of autophagy, inhibition of NLRP3 and TNF-α, alleviation of ER stress, and suppression of ROS via mitochondria function improvement. Whether these effects of GLP-1RAs on the liver are indirect or some of them may be direct, i.e. through activating GLP-1R on hepatocytes is still a controversial issue, since the presence of GLP-1R in the liver has been challenged. Undoubtedly, the well-established action of GLP-1RAs in extrahepatic tissues (i.e., brain, stomach, pancreas, and gut) indirectly affects the liver in a positive manner

Most animal studies demonstrated a beneficial effect of GLP-1RAs and related peptide polyagonists on LFTs, glucose, and lipid metabolism and, most importantly, on hepatic steatosis and inflammation (Tables 1 and 2). However, the effect of GLP-1RAs on hepatic fibrosis seems to be minimal, which is in line with major clinical trials on the effect of liraglutide [49] and semaglutide [50] in patients with NASH. More specifically, both RCTs with paired liver biopsies showed improvement in hepatic steatosis and inflammation, but not fibrosis, after treatment with liraglutide or semaglutide. Although few animal studies reported an improvement in indices of fibrosis or prevention of the initiation of fibrosis [8, 26, 29], most studies do not support a robust effect of GLP-1RAs on hepatic fibrosis (Table 1). A summary of the multiple mechanisms through which GLP-1RAs have been hypothesized to beneficially affect NAFLD based on animal studies are depicted in Fig. 1. By contrast, the initial studies on GLP-1RA related peptide polyagonists in animal models of NAFLD have to date provided more encouraging results as regards their effect on hepatic fibrosis (Table 2). Notably, in some of them, the effect of the polyagonist was superior to GLP-1RAs [45, 46].

Considering the above and given that hepatic fibrosis is the main histological prognostic factor for advanced NAFLD and a target that is difficult to reverse [51], it may be speculated that GLP-1RAs could possibly be provided in earlier stages of the disease before the occurrence of hepatic fibrosis; in this case, by improving hepatic steatosis and inflammation, GLP-1RAs may prevent or delay the fibrogenic progress of the disease. However, this remains to be definitively shown; in this regard, animal studies with early administration of GLP-1RA and long-term follow-up are needed, since fibrosis is a longitudinal progress, before this hypothesis is investigated in relevant clinical trials with NAFLD patients without significant hepatic fibrosis. This is in contrast to most previous and ongoing clinical trials in which patients with NASH and significant or advanced fibrosis were included and largely failed to show improvement in fibrosis [5]. However, based on animal studies with GLP-1RA related peptide polyagonists, they seem to be more effective in improving hepatic fibrosis, possibly due to the additive or synergistic effects of more than one receptor agonist. In this regard, the setting of clinical trials focusing on the improvement of established hepatic fibrosis after treatment with peptide polyagonists seems to be more rational. Currently, there are few ongoing clinical trials with peptide polyagonists in NASH, including tirzepatide (SYNERGY-NASH; NCT04166773), efinopegdutide (NCT05877547), and cotadutide (PROXYMO-ADV; NCT05364931), as elsewhere summarized [41].

Apart from the combinations of GLP-1RAs with other incretin analogs, it should be highlighted that a GLP-1RA was combined with an FGF-21 analog in a single molecule (GLP-1-Fc-FGF21 D1), yielding encouraging results [48]. Indeed, clinical trials with FGF-21 analog monotherapy (e.g., pegbelfermin, efruxifermin, and BOS-580) have generated favorable results in NAFLD, as elsewhere summarized [52]. Therefore, the combination of a GLP-1RA and an FGF-21 analog in the same molecule seems to be an appealing approach; however, further studies in different animal models are required to achieve more secure results and, importantly, to elucidate the effect of GLP-1RA/FGF-21 analog on hepatic fibrosis.

A relevant issue that remains controversial is whether the positive impact of GLP-1RAs on hepatic histology is indirect, i.e., through their action on the pancreas or other extrahepatic tissues, or direct, i.e., through activating GLP-1R on hepatocytes. GLP-1R is a G protein-coupled receptor, whose visualization is impeded by its relatively low abundance and the lack of specific and validated antibodies for its detection that reliably work [53]. Some authors have supported the presence of GLP-1 receptors on the hepatocytes [54], while others do not [55]. It has also been supported that a small fragment of GLP-1 may also enter the hepatocytes, thus targeting mitochondria and exerting insulin-like actions, which, however, needs further validation [56]. The receptors of other peptide agonists may be present at the hepatocyte, e.g., GCG receptor, through which cotadutide was supported to have direct hepatic effects [45]. However, much work is still needed on this topic.

As mentioned above, the nomenclature NAFLD has been changed, first to metabolic (dysfunction)-associated fatty liver disease (MAFLD) [57] and more recently to metabolic dysfunction-associated steatotic liver disease (MASLD) [2]. The change in the name is accompanied by changes in the definition of the disease, which has opened up a global discussion on the topic [58]. Since NAFLD, MAFLD, and MASLD are very closely related but not identical entities, data on NAFLD should be carefully extrapolated to MAFLD or MASLD. Therefore, we preferred to keep the original nomenclature of NAFLD in this review, since all included animal studies referred to NAFLD.

In conclusion, most animal studies support a beneficial effect of GLP-1RAs on NAFLD histology, including steatosis and inflammation, but not on fibrosis, while a few initial animal studies with GLP-1RA related polypeptides have provided limited evidence for a more favorable effect of peptide polyagonists on hepatic fibrosis. However, the effect of peptide polyagonists needs further exploration in animal studies as well as in mechanistic studies to investigate the potentially additive or synergistic effect of different receptor agonists, and their specific mechanisms of action. Research on the management of NAFLD is an imperative need due to its high prevalence, morbidity, and mortality and the concomitant lack of any specifically approved medication. In this regard, relevant animal studies are regarded as highly important.

## Data Availability

Since this is a narrative review, no raw data were generated specifically for this manuscript.

## Abbreviations

*AMLN*: Amylin liver nonalcoholic steatohepatitis
*ER*: Endoplasmic reticulum
*FFA*: free fatty acids
*FFD*: Fast-food diet
*GAN*: Gubra amylin
*GLP-1RAs*: Glucagon-like peptide-1 receptor agonists
*HFCC*: High-fat high-cholesterol high-cholic acid
*HFCC-CDX*: High-fat high-cholesterol high-cholic acid diet with hydroxypropyl-β-cyclodextrin in drinking water
*HFD*: High-fat diet
*HTFD*: High-trans-fat high-fructose high-cholesterol diet
*MCD*: Methionine-choline deficient
*NAFLD*: Nonalcoholic fatty liver disease
*NLRP3*: Nod-like receptor protein amilypyrin domain containing 3
*ROS*: Reactive oxygen species
*SD*: Sprague-Dawley
*TNF-α*: Tumor necrosis factor-α
